# Effects on mouse melanoma and immune cells of P2X7 receptor positive allosterism by clemastine

**DOI:** 10.1007/s11302-026-10179-x

**Published:** 2026-08-28

**Authors:** Maria Luiza Thorstenberg, Simonetta Falzoni, Rocio Edith Garcia-Jacobo, Leticia Scussel Bergamin, Mario Tarantini, Giulia Conversano, Anna Pegoraro, Francesco Di Virgilio, Elena Adinolfi, Anna Lisa Giuliani

**Affiliations:** Department of Medical Sciences, University of Ferrara, Via Luigi Borsari, 46, Ferrara, 44121 Italy

**Keywords:** P2X7 receptor, Clemastine, B16F10 melanoma cells, Macrophages, IL-1β

## Abstract

Clemastine (CLE), an H1R antagonist widely used for the treatment of allergic rhinitis, has recently been investigated for its positive modulatory effects on P2X7, a receptor for extracellular ATP (eATP) involved in the regulation of inflammation, immune responses, and the composition of the tumor microenvironment (TME). Here, we further examined the effects of CLE on P2X7-mediated responses to clarify the pharmacological significance of P2X7 potentiation. First, in murine and human cells expressing (B16F10 WT and HEK293-hP2X7) or lacking (B16F10 P2X7^−/−^ and HEK293 WT) the P2X7 receptor, we measured intracellular Ca^2+^ influx, ethidium bromide (EB) and daunorubicin (DN) uptake, mitochondrial morphology, and LDH and ATP release following P2X7 agonism or antagonism in the presence of CLE. We found that CLE increased Bz-ATP-induced P2X7-mediated channel opening, pore formation, mitochondrial remodeling, eATP release, and cell death, effects absent in P2X7-null cells and abolished by P2X7 antagonists. As a readout of activation of NLRP3 inflammasome, a known downstream effector of the P2X7 receptor, we also assessed the effect of CLE on IL-1β release from peritoneal macrophages from wild-type (WT) or P2X7 knockout (KO) mice, LPS-primed and treated with ATP and/or CLE. We found that the combined treatment significantly increased IL-1β release from P2X7-WT macrophages compared to the two stimuli administered separately. On the whole, our results demonstrate that CLE can effectively positively modulate P2X7 function, suggesting new opportunities for its therapeutic exploitation.

**Supplementary information:**

The online version contains supplementary material available at https://doi.org/10.1007/s11302-026-10179-x.

## Introduction

Adenosine 5′-triphosphate (ATP) can be released into the extracellular space, where it acts as a potent danger signal and canonical damage-associated molecular pattern (DAMP). High extracellular ATP (eATP) concentrations are detected at sites of tissue injury, inflammation, and within the tumor microenvironment (TME) [1, 2]. In these contexts, eATP triggers immune cell recruitment and activation, promotes cytokine release, and modulates stromal and tumor cell activities [3, 4]. The magnitude and duration of eATP exposure critically influence downstream responses, as ATP bursts trigger acute inflammation, while sustained elevated levels contribute to chronic immune dysfunction within the TME [5].

The effects of eATP are primarily mediated by purinergic receptors (P2Rs), a heterogeneous family of plasma membrane receptors specialized in detecting extracellular nucleotides. P2Rs are divided into two main subfamilies: the G protein-coupled P2Y receptors, which activate intracellular signaling pathways via Gi or Gq proteins, and the ligand-gated P2X receptors, which mediate rapid ion fluxes across the plasma membrane [6]. The P2X receptor family includes seven isoforms (P2X1–P2X7), each characterized by distinct ATP sensitivity, kinetics, and ligand modulation [7, 8].

Among P2X receptors, P2X7 is functionally unique. Upon activation by ATP or by the potent agonist 2′(3′)-O-(4-benzoylbenzoyl) adenosine 5′-triphosphate (Bz-ATP), P2X7 allows Ca^2^⁺ and Na⁺ influx together with K⁺ efflux. Prolonged exposure to elevated eATP concentrations triggers the opening of a large, nonselective macropore that permits the passage of molecules up to approximately 900 Da. P2X7 activation can trigger membrane blebbing, inflammasome activation, and cell death [9].

A key downstream consequence of P2X7 activation is the engagement of the NLRP3 inflammasome. The ionic perturbations triggered by the receptor—particularly K⁺ efflux—promote NLRP3 assembly and caspase-1 activation, leading to the maturation of IL-1β and IL-18 and, in some contexts, to pyroptotic cell death [10, 11]. In pathological settings, such as tumors, characterized by continuous eATP release, sustained NLRP3 activation downstream of P2X7 contributes to shaping the TME [12, 13]. P2X7 is frequently overexpressed in solid tumors and hematologic malignancies, contributing to enhanced tumor growth, invasiveness, metastatic potential, and poor prognosis [9, 14, 15]. P2X7 influences cytokine release, immune cell activation, and tumor cell survival, highlighting its relevance as a promising pharmacological target in oncology [3, 16]. Despite the substantial body of evidence linking P2X7 overexpression to tumor growth and progression [3], the cytotoxic potential of this receptor, arising from its ability to form a membrane macropore [9], has led several groups to propose an alternative therapeutic strategy based on positive allosteric modulators (PAMs) to promote the selective death of tumor cells that overexpress P2X7 [17–20]. Indeed, several compounds are capable of positively modulating this receptor, and some of them have demonstrated antitumor efficacy in combination with anti-PD-1 inhibitors [21]. Among the PAMs of P2X7 identified to date are endogenous molecules such as β-amyloid [22] and LL-37 [23], plant-derived compounds such as ginsenosides [24, 25], and synthetic agents including tenidap [26], polymyxin B [27, 28], HEI3090 [21], and clemastine (CLE) [29].

Among the known PAMs, CLE stands as a valuable tool for dissecting the allosteric regulation of P2X7, and, as an FDA-approved antihistamine drug with well-defined pharmacokinetics and a favorable safety profile, it could offer an opportunity to explore the physiological and therapeutic implications of P2X7 potentiation [30]. Given the central involvement of P2X7 in inflammation, immunity, and tumor biology, in this study, we investigated whether CLE acts as a PAM of P2X7 in both tumor and immune cells. Specifically, we assessed its effects on channel and macropore formation, mitochondrial and cell damage or death, and IL-1β release to evaluate its potential relevance in inflammatory and cancer-related contexts. Through this approach, we aimed to clarify the pharmacological significance of P2X7 potentiation and identify new opportunities for its therapeutic exploitation.

## Materials and methods

### Reagents

Clemastine fumarate (CLE) (Merck, cat. no. SML0445), 2′,3′-O-(4-benzoylbenzoyl)-adenosine 5′-triphosphate (Bz-ATP) (Merck, cat. no. B6396), A740003 (Tocris, cat. no. 3701/10), ethidium bromide (EB) (Merck, cat. no. E8751), daunorubicin hydrochloride (Merck, cat. no. 23541-50−6), Fura-2 AM (Invitrogen, cat. no. F1201), MitoTracker Green (Thermo Fisher, cat. no. M7514), bovine serum albumin (BSA) (Merck, cat. no. A9418), fetal bovine serum (FBS) (Invitrogen, cat. no. 15140130), oATP (Sigma, cat. no. A23836), polymyxin B (PB) sulfate salt (Sigma, cat. no. P4932), 8% Bolt™ Bis-Tris Plus gel, 1.0 mm, WedgeWell™ format (Invitrogen, cat. no. NW00082 BOX), NuPage LDS (Invitrogen, cat. no. NP0007), nitrocellulose membrane (Bio-Rad, cat. no 1620115), and protease inhibitor cocktail (Sigma cat. no. P8849) were used.

### Cell cultures

B16F10 WT and B16F10 P2X7^⁻/⁻^ cells (originating from C57Bl/6j background, obtained from ATCC®CRL-6475) were cultured in RPMI-1640 medium (Sigma-Aldrich, cat. no. R0883) supplemented with 10% heat-inactivated FBS, 100 U/mL penicillin, and 100 µg/mL streptomycin. B16F10 P2X7^⁻/⁻^ cells were maintained under continuous selection with puromycin dihydrochloride (0.8 µg/mL, Thermo Fisher, cat. no. A1113803). HEK293 cells were maintained in DMEM/F12 (Sigma-Aldrich, cat. no. D666421) supplemented with 10% heat-inactivated FBS. Stable hP2X7-transfected HEK293 human clones (HEK293 hP2X7) previously obtained in our laboratory [31] were cultured in the presence of 200 µg/mL G418 sulfate (Geneticin, Calbiochem, cat. no. 509290). Peritoneal macrophages were harvested from WT and P2X7 KO C57Bl/6 mice by peritoneal lavage with cold phosphate-buffered saline (PBS). The collected cells were seeded into 24-well culture plates, in RPMI 1640 medium (10% FBS, 1% L-glutamine, 1% P/S) and incubated at 37 °C with 5% CO_2._ After 1 h, the cultures were gently washed twice with PBS to remove nonadherent cells and treated as described below.

### CRISPR/Cas9 P2X7 gene knockout

The CRISPR/Cas9 P2X7 gene knockout in B16/F10 cells was obtained by the commercially available kit Double Nickase Plasmid (m) sc-422093-NIC and Control Double Nickase Plasmid sc-437281, according to the manufacturer’s (Santa Cruz Biotechnology Inc) instructions.

Guide RNAs (forward: 5′-CTGACCGGCGTTGTAAAAAG-3′; reverse: 5′-GAGCACTGTGCACCGCGCCT-3′) were designed to target an intragenic region spanning exons 2 and 3 of Gene ID 18439. These sequences correspond to the amino acid motifs DRRCKK (aa 132–137) and RRGAQC (aa 124–129), respectively, of Ref Seq NM_011027.4.

Transfection was performed with TransIT-2020 transfection reagent (Tema Ricerca, Bologna, Italy), and the transfected cells were maintained in antibiotic selection with puromycin 0.8 µg/mL. After 48 h, single-cell clones were isolated by limiting dilution and expanded for approximately 5 weeks. Successful gene knockout was assessed by western blot.

### Western blot

Total proteins were extracted from B16F10 WT and B16P2X7^−/−^ using RIPA lysis buffer supplemented with a 1 × protease inhibitor cocktail. Protein concentration was determined using the Bradford assay. Equal amounts of protein (20 µg or 15 µg) were separated using an 8% Bolt™ Bis–Tris Plus gel or a 4–12% NuPAGE™ Bis–Tris gel (Invitrogen) and subsequently transferred onto a nitrocellulose membrane. To minimize nonspecific binding, membranes were blocked with 3% bovine serum albumin (BSA) in TBS-t (0.05%) for 1 h at room temperature. Membranes were then incubated overnight at 4 °C with rabbit polyclonal anti-P2X7 receptor antibodies directed against either the extracellular domain (Alomone Labs, cat no. APR-008) or the C-terminal domain (Sigma cat no. P8232), diluted 1:500 in 3% nonfat dry milk and 0.5% BSA, or with a rabbit polyclonal anti β-actin antibody (Sigma, cat. no A5060) diluted 1:2000 in 5% nonfat dry milk. Following three 10-min washes with TBS-t, membranes were incubated with a horseradish peroxidase (HRP)–conjugated goat anti-rabbit IgG antibody (Invitrogen, cat. no. A16110) diluted 1:2000 in 5% nonfat dry milk for 1 h at room temperature. Protein bands were visualized by an enhanced chemiluminescence (ECL) detection system (Cyanagen, cat. no. XLS0710250) after keeping the membrane in the dark in contact with substrate. Images were captured with a ChemiDoc™ Imaging System (Bio-Rad) or with a LI-COR blot scanner (Biosciences, Lincoln, NE, USA). Densitometry was performed using Image Lab Software (ChemiDoc system) or ImageJ (Li-COR system). The P2X7 receptor was identified as a characteristic band at approximately 68–75 kDa, representing the mature protein, while β-actin was used as the internal loading control. Images of representative western blots are provided in the supplementary information.

### Intracellular Ca^2^⁺ measurements

Variations in intracellular Ca^2^⁺ concentration ([Ca^2^⁺]ᵢ) were assessed with Fura-2 AM using an LS50 fluorometer (PerkinElmer, Shelton, USA). B16F10 WT, B16F10 P2X7^−/−^, HEK293 WT, and HEK293-hP2X7 cells (1.0 × 10⁶/mL) were resuspended in saline solution (125 mM NaCl, 5 mM KCl, 1 mM MgSO_4_, 1 mM NaH_2_PO_4_, 20 mM HEPES, 5.5 mM glucose, 5 mM NaHCO_3_, and 1 mM CaCl_2_, pH 7.4) and loaded for 20 min at 37 °C with 4 µM Fura-2 AM and 250 µM sulfinpyrazone to ensure intracellular Fura-2AM retention. After washing, cells were placed into a stirred fluorometric cuvette at 37 °C and treated sequentially with A740003 (10 µM), CLE (25 µM), and/or Bz-ATP (300 µM). Fluorescence ratios (340/380 nm excitation, 505 nm emission) were recorded. Results are expressed as representative traces and as areas under curve (AUCs).

### Ethidium bromide (EB) uptake assay

P2X7-dependent pore formation was assessed by EB uptake. B16F10 WT and B16F10 P2X7^−/−^ cells (1.0 × 10⁶/mL) were resuspended in sucrose saline (30 mM sucrose, 1 mM K_2_HPO_4_, 1 mM MgSO_4_, 5.5 mM glucose, 20 mM HEPES, pH 7.4), placed in a stirred fluorometric cuvette at 37 °C in the presence of EB (20 µM) and treated sequentially with A740003 (10 µM), CLE (25 µM), and/or Bz-ATP (300 µM). Digitonin (100 µM) was added at the end of each experiment to obtain maximal fluorescence (100% permeabilization). EB fluorescence was recorded at 360 nm excitation and 580 nm emission using an LS50 fluorometer (PerkinElmer).

### Daunorubicin (DN) uptake assay

B16F10 WT cells (2.0 × 10^5^) were plated onto 35-mm dishes (Euroclone®, cat. no. ET2035) and imaged on a Leica DMI 4000B (Leica Microsystems CMS GmbH) microscope. Cells were incubated in saline solution supplemented with DN (1 µg/mL). CLE (25 µM) and Bz-ATP (300 µM) were then added sequentially to evaluate macropore-dependent DN uptake. After 30 min of incubation, DN fluorescence was recorded at 328 nm excitation and 545 nm emission. ImageJ (ImageJ 1.53t/Java 1.8.0) was used to quantify fluorescence. The percentage of positive cells was normalized to number of cells obtained from phase-contrast images.

### MitoTracker Green staining

B16F10 WT cells (2.0 × 10^4^) were cultured onto 24-mm glass coverslips previously coated with a 0.1% poly-L-lysine solution and cultured for 24 h. Cells were then incubated with MitoTracker™ Green FM (200 nM) for 30 min at 37 °C in RPMI-1640. Live-cell imaging was performed using an Olympus FV3000 confocal microscope equipped with FV31S-SW software (Olympus, Tokyo, Japan), exciting the fluorophore at 488 nm. Baseline images (*t* = 0) were captured prior to sequential treatments with A740003 (10 µM), CLE (25 µM), and Bz-ATP (500 µM), and confocal micrographs were collected exactly after 15 min of incubation with each compound. All images were subsequently processed and analyzed using MetaMorph and ImageJ/Fiji software. Confocal images of MitoTracker Green fluorescence were processed, binarized, and subjected to particle analysis to generate mitochondrial morphological characteristics for each cell: aspect ratio (AR, ratio between the major and minor axis of the ellipse equivalent to the mitochondrion, where AR = 2.0 indicates longer and tubular mitochondrial network) and degree of circularity (where a value of 1.0 indicates a perfect sphere) using standardized particle analysis protocols [32, 33].

### LDH assay

To evaluate the cytotoxicity of CLE at different concentrations and estimate its optimal working concentration, B16F10 WT cells or mouse peritoneal macrophages were seeded into 24-well plates and treated overnight with CLE (10–100 µM). In a separate set of experiments, B16F10 WT cells (2.0 × 10^4^ cells/well) were treated overnight with A740003 (10 µM), CLE (25 µM), and/or Bz-ATP (300 µM), added sequentially in this specific order. Alternatively, B16F10 WT cells were pretreated for 2 h with oATP (300 µM), followed by the addition of polymyxin B (PB) (10 µg/mL) and Bz-ATP (300 µM) in a subsequent overnight incubation time. After the treatments, plates were centrifuged for 5 min at 1100 rpm and supernatants were collected for LDH quantification using the CytoQuant LDH Cytotoxicity Assay Kit (Invitrogen, cat. no. C20301) according to manufacturer’s instructions. Maximum LDH release was determined lysing control untreated cells (positive control) whereas culture medium served as negative control. Absorbance was measured at 490 nm and 680 nm using a Victor 3™ fluorometer (PerkinElmer). Cytotoxicity (%) = (test−negative control)/(positive control−negative control) × 100.

### ATP measurement

B16F10 WT cells (8 × 10^3^ cells) were seeded into 96-well dark plates (Greiner, cat. no. 655090) for 24 h in complete RPMI-1640 medium. At the end, cells were treated for 30 min with CLE (25 µM) and Bz-ATP (300 µM). Extracellular ATP levels were quantified at a Victor 3™ fluorometer (PerkinElmer) using the ENLITEN Luciferase/Luciferin assay (Promega, cat. no. FF2021) following the manufacturer’s instructions as previously described [34]. CPS (counts per second) were converted to ATP concentration (nM) using an ATP standard curve.

### IL-1β release and measurement by ELISA

Peritoneal macrophages were obtained from WT and P2X7 KO C57Bl/6 mice (authorization n. 264/2021-PR from the Italian Ministry of Health). Three × 10^5^ cells were seeded for 1 h onto glass coverslips, washed three times with PBS, and cultured in complete DMEM (10% FBS, 1% L-glutamine, 1% P/S). Adherent macrophages were primed for 2 h with LPS (1 µg/mL) and stimulated for 30 min with CLE (20 µM) and/or ATP (500 µM). Stimulation with 3 mM ATP alone was used to obtain the maximal IL-1β release. Supernatants were collected and, after centrifugation, IL-1β levels were measured using a mouse IL-1β ELISA kit (R&D Systems, cat. no. MLB0000C-1), following the manufacturer’s instructions. Absorbance was measured at 450 nm, and cytokine concentrations were expressed in pg/mL.

### Statistical analysis

GraphPad Prism 11.0 software (San Diego, CA, USA) was used for statistical analysis and graphing. Data are presented as mean ± SEM. Two-group comparisons were performed using Student’s *t* test. For experiments with more than two groups, one-way ANOVA followed by Tukey’s post hoc test was applied. Results were considered statistically significant when *p* < 0.05.

## Results

### CLE upregulates P2X7-dependent ion channel and macropore formation

P2X7 can be positively modulated by different molecules among which CLE, as previously shown [29]. To verify if these findings could be replicated in different cell types, we investigated responses to CLE plus P2X7 agonist in mouse melanoma B16F10 WT and in human HEK293-hP2X7 cells. As negative controls, we used either B16F10 P2X7^-/-^ cells, in which the *p2x7* gene was knocked out by CRISPR/Cas9 technique (see Materials and methods) (Supplementary Figure 1), or HEK293 WT cells, which do not express P2X7. Unless otherwise specified, the stimuli here below were used at the following concentrations: 300 µM Bz-ATP, 25 µM CLE, and 10 µM A740003. The concentrations of CLE used here and in subsequent experiments were established based on cytotoxicity assessment at various dosages on different cell types (Supplementary Figure 2).

**Fig. 1:**
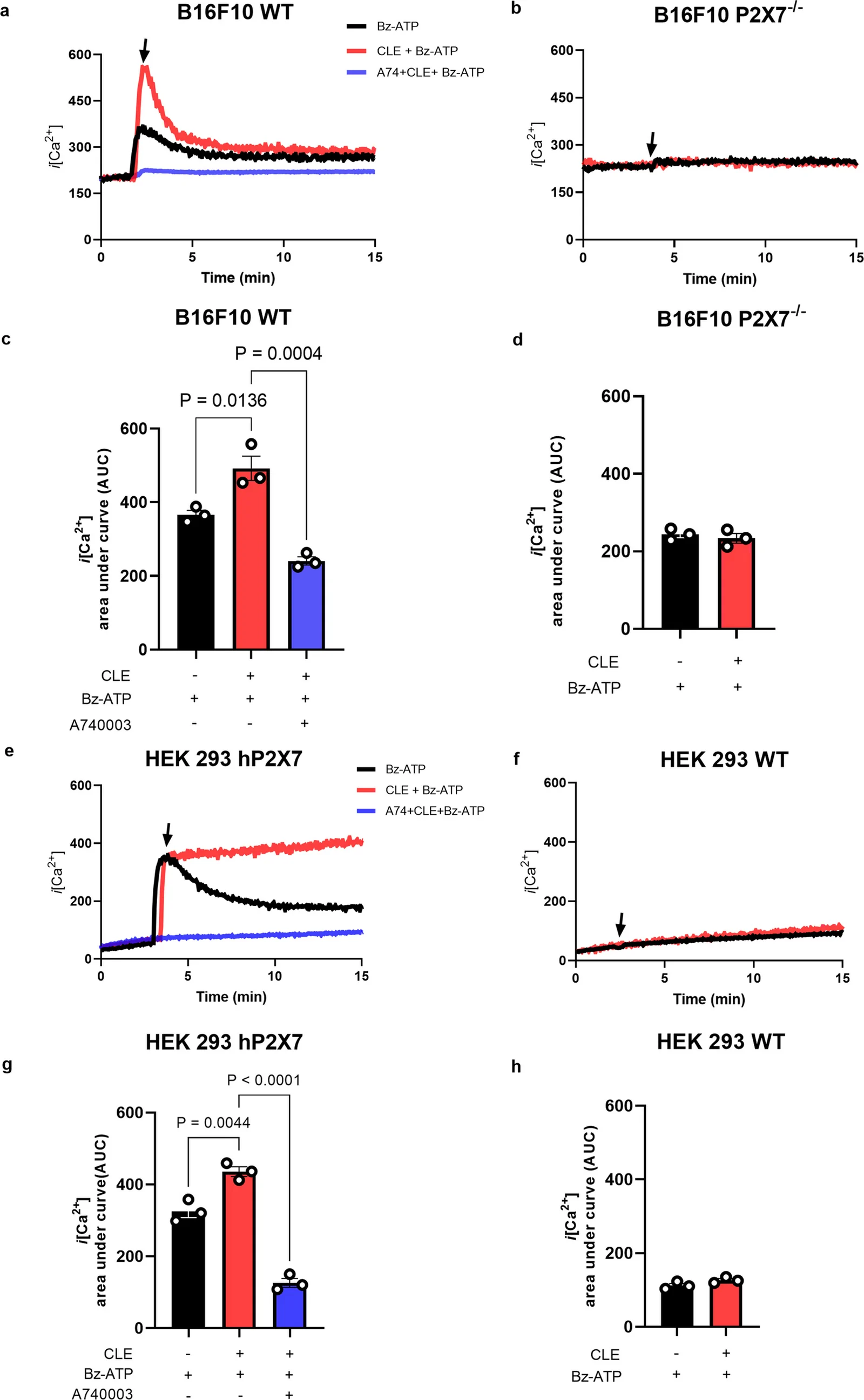
CLE increases P2X7-dependent channel opening in murine tumor and human cells. **a**, **c** B16F10 WT, **b**, **d** B16F10 P2X7^−/−^, **e**, **g** HEK293-hP2X7, and **f**, **h** HEK293 WT cells (1 × 10⁶ cells/mL) were incubated for 20 min at 37 °C with Fura-2 AM (4 µM). **a**, **b**, **e**, **f** Representative traces of [Ca^2^⁺]ᵢ changes measured as described under the methods induced by sequential treatment with CLE (25 µM) and/or Bz-ATP (300 µM). Where indicated, the P2X7 antagonist A740003 (10 µM) was added to the cells prior to the other compounds. Arrows indicate the time of stimulation. **c, d, g**, **h** Quantification of [Ca.^2^⁺]ᵢ changes as area under the curve (AUC). Data are expressed as mean ± SEM from three independent experiments. Statistical analysis was performed using one-way ANOVA followed by Tukey’s multiple comparisons test. Results were considered statistically significant when *p* < 0.05

First, we evaluated P2X7-dependent ion channel formation by measuring [Ca^2^⁺]ᵢ changes (Fig. 1). As expected, stimulation with Bz-ATP increased [Ca^2^⁺]ᵢ in P2X7-expressing (Fig. 1a, c, e, g) but not in P2X7-lacking cells (Fig. 1b, d, f, h). In cells expressing the P2X7 receptor, sequential treatment with CLE and Bz-ATP caused a significantly higher increase of [Ca^2^⁺]ᵢ than treatment with Bz-ATP alone (Fig. 1a, c, e, g) and the P2X7 antagonist A740003 significantly reduced this effect (Fig. 1a, c, e, g).

**Fig. 2:**
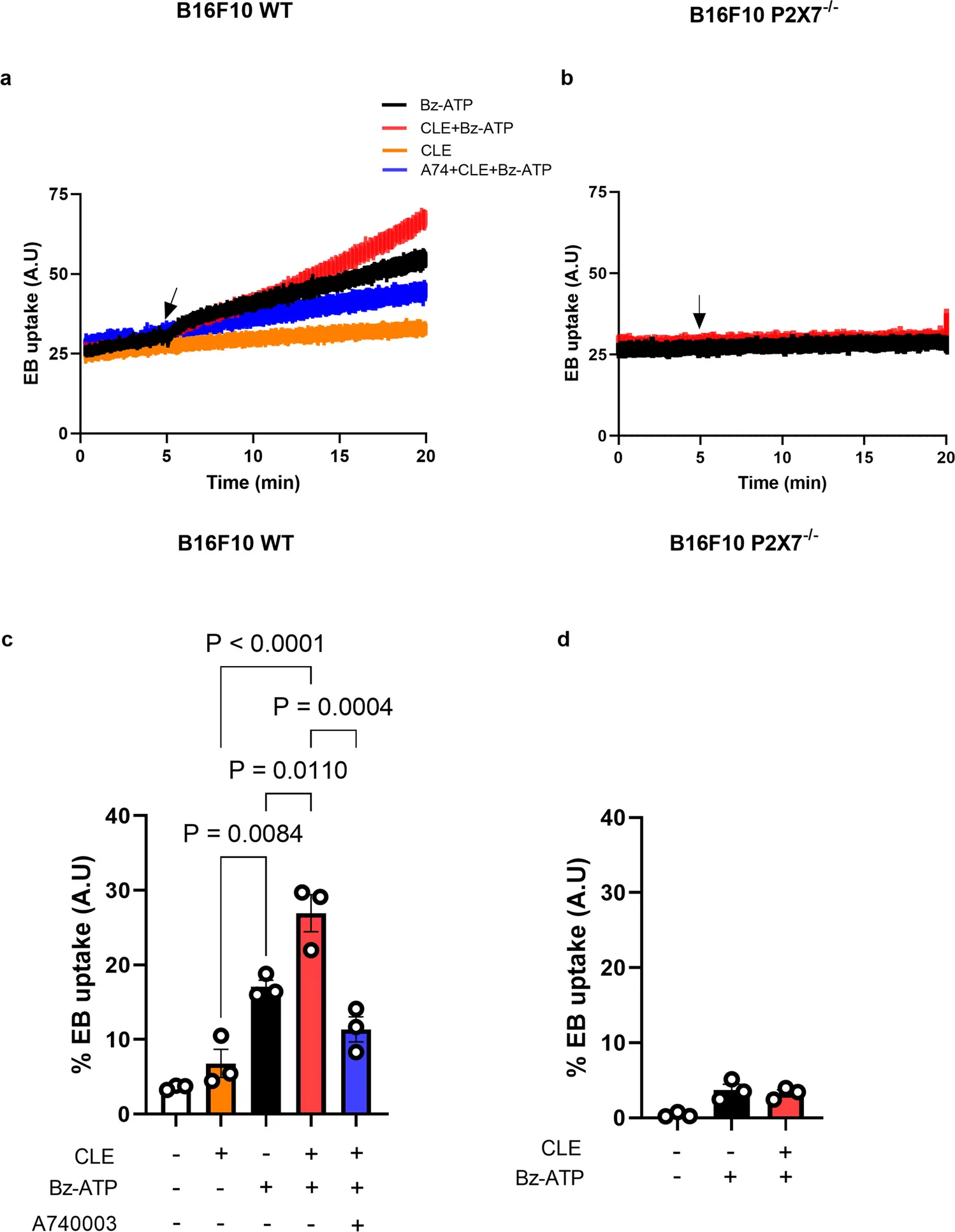
CLE increases P2X7-dependent pore formation in murine tumor cells. **a**, **b** Representative traces of ethidium bromide (EB) uptake measured as described under methods, induced by sequential treatment with CLE (25 µM) and/or Bz-ATP (300 µM), in **a** B16F10 WT and **b** B16F10 P2X7^⁻/⁻^ cells. Where indicated, the P2X7 antagonist A740003 (10 µM) was added to the cells prior to the other compounds. Arrows indicate the time of stimulation. **c**, **d** Quantification of EB uptake is expressed as percentage of digitonin-induced maximal uptake (set as 100%) in **c** B16F10 WT and **d** B16F10 P2X7^⁻/⁻^ cells. Data are expressed as mean ± SEM from three independent experiments. Statistical analysis was performed using one-way ANOVA followed by Tukey’s multiple comparisons test. Results were considered statistically significant when *p* < 0.05

We next evaluated ethidium bromide (EB) uptake as a readout of increased plasma membrane permeability due to P2X7-dependent macropore formation. In B16F10 WT cells, sequential treatment with CLE and Bz-ATP significantly increased EB uptake compared to treatment with Bz-ATP alone, while the P2X7 antagonist A740003 significantly reversed this result (Fig. 2a, c). EB entry induced by CLE and Bz-ATP required P2X7 expression, since it was lost in B16F10 P2X7^⁻/⁻^ cells (Fig. 2b, d).

### CLE enhances P2X7-dependent daunorubicin (DN) uptake in murine tumor cells

**Fig. 3:**
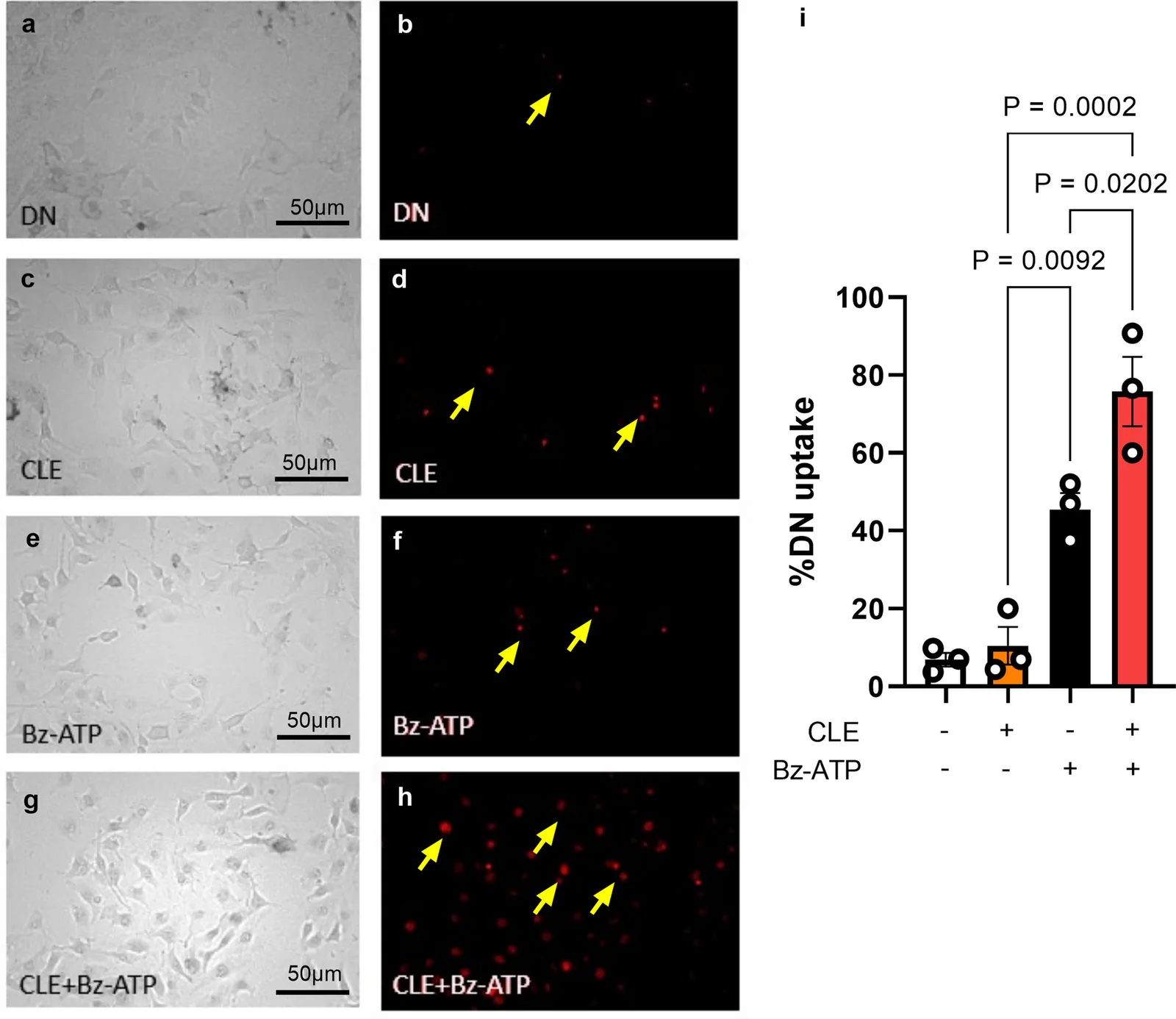
CLE enhances P2X7-dependent daunorubicin (DN) uptake in murine tumor cells. B16F10 WT cells (2.0 × 10^5^) were seeded onto 35-mm dishes and incubated with DN (1 µg/mL) in sodium saline solution. Total number of cells/field was counted using phase-contrast images (**a**, **c**, **e**, **g**), and number of DN-positive cells/field was obtained analyzing fluorescence images after 30 min of sequential treatment with CLE (25 µM) and/or Bz-ATP (300 µM) (**b**, **d**, **f**, **h**). DN uptake was quantified as the percentage of DN-positive cells (**i**), calculated using the formula: (number of DN−positive cells/total number of cells) × 100. Data represent the mean ± SEM of three independent experiments. Scale bars are displayed in all phase contrast photographs. Statistical analysis was performed using one-way ANOVA followed by Tukey’s multiple comparisons test. Results were considered statistically significant when *p* < 0.05

Since P2X7 macropore opening has been shown to facilitate the uptake of the chemotherapeutic drug daunorubicin (DN) leading to cell death in acute myeloid leukemia cells [35], we investigated whether CLE treatment could enhance DN uptake into melanoma cells as a consequence of increased P2X7-dependent macropore formation. As shown in Fig. 3, the number of DN-associated red fluorescent nuclei was indeed significantly higher in B16F10 WT cells treated sequentially with CLE and Bz-ATP than in cells treated with Bz-ATP alone.

### CLE increases P2X7-dependent mitochondrial alterations and cell death

P2X7 stimulation with maximal doses of agonists has been shown to shift the receptor from exerting trophic effects to inducing mitochondrial network disruption [36]. This is not surprising, given that the P2X7 receptor has recently been found in mitochondria themselves [37, 38]. Therefore, we analyzed mitochondrial morphological changes in B16F10 WT melanoma cells cultured onto 40-mm glass coverslips for 24 h and then stained for 30 min with MitoTracker Green FM (200 nM). The mitochondrial network was evaluated after 15 min of sequential incubation with CLE (25 µM) and/or Bz-ATP (500 µM). Where indicated, A740003 (10 µM) was added to the cells prior to the other reagents.

**Fig. 4:**
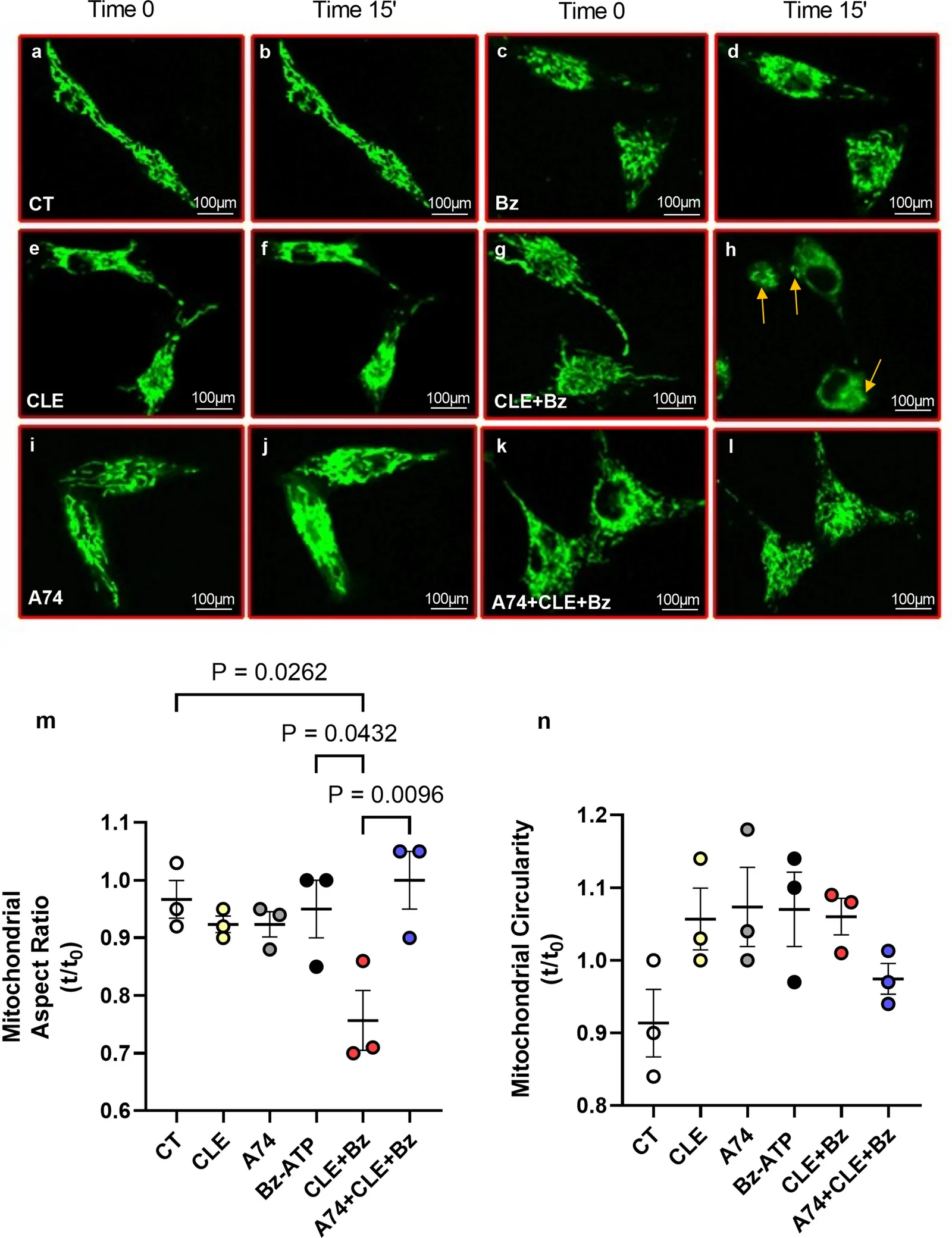
CLE increases P2X7-dependent mitochondrial structural alterations. B16F10 WT cells (2.0 × 10^4^) were seeded onto 40-mm glass coverslips and cultured for 24 h in complete RPMI 1640 medium. Cells were then stained with MitoTracker Green (200 nM) for 30 min in RPMI culture medium. Live-cell imaging was performed using an Olympus FV3000 confocal microscope equipped with FV31S-SW software (Olympus, Tokyo, Japan), exciting the fluorophore at 488 nm. Baseline images (*t* = 0) were captured prior to sequential treatments with A740003 (10 µM), CLE (25 µM), and Bz-ATP (500 µM), and confocal micrographs were collected exactly after 15 min of incubation with each compound. All images were subsequently processed and analyzed using MetaMorph and ImageJ/Fiji software. **c**, **d** BzATP (500 µM). **e**, **f**, CLE (25 µM). **g**, **h** CLE + Bz-ATP. **i**, **j** A740003 (10 μM). **k**, **l** A740003 + CLE + Bz-ATP. Images are representative of three separate experiments. Arrows indicate mitochondrial damage areas. Quantification of **p** aspect ratio (AR) (a measure of mitochondrial elongation) and **q** circularity (where a value close to 1 indicates a perfect sphere) by digital image analysis by standardized particle analysis protocols in ImageJ/Fiji on the high-resolution confocal micrographs obtained from multiple independent biological replicates as described in the “ Materials and methods” section

At baseline (time zero), the probe enabled clear visualization of the mitochondrial network (Fig. 4a, c, e, g, i, k). Treatment for 15 min with Bz-ATP or CLE separately induced mild mitochondrial modifications (Fig. 4d, f), while costimulation dramatically increased these structural alterations (Fig. 4h) which were reduced by the P2X7 antagonist A740003 (Fig. 4l). The morphometric data confirmed our visual observations. Under control conditions, mitochondria exhibited a high aspect ratio (AR) and low circularity, typical of an interconnected, tubulated network. AR values decreased significantly following treatment with CLE and Bz-ATP and returned to levels comparable to those of the control in cells pretreated with A740003 (Fig. 4m). Conversely, the increase of mitochondrial circularity following CLE and Bz-ATP treatment was not significant and the A740003 pretreatment only partially reversed this increase (Fig. 4n). This quantitative shift objectively validates the transition from elongated networks to altered, circular mitochondrial structures.

Since the opening of the P2X7 macropore was also associated with cell death, we measured LDH release in the supernatants of cells stimulated with CLE and/or Bz-ATP. B16F10 WT cells were treated overnight with A740003 (10 µM), CLE (25 µM), and/or Bz-ATP (100 µM). Alternatively, cells were pretreated for 2 h with oATP (300 µM) and then polymyxin B (PB) (10 µg/mL), and Bz-ATP (100 µM) was added to cell cultures as described above.

**Fig. 5:**
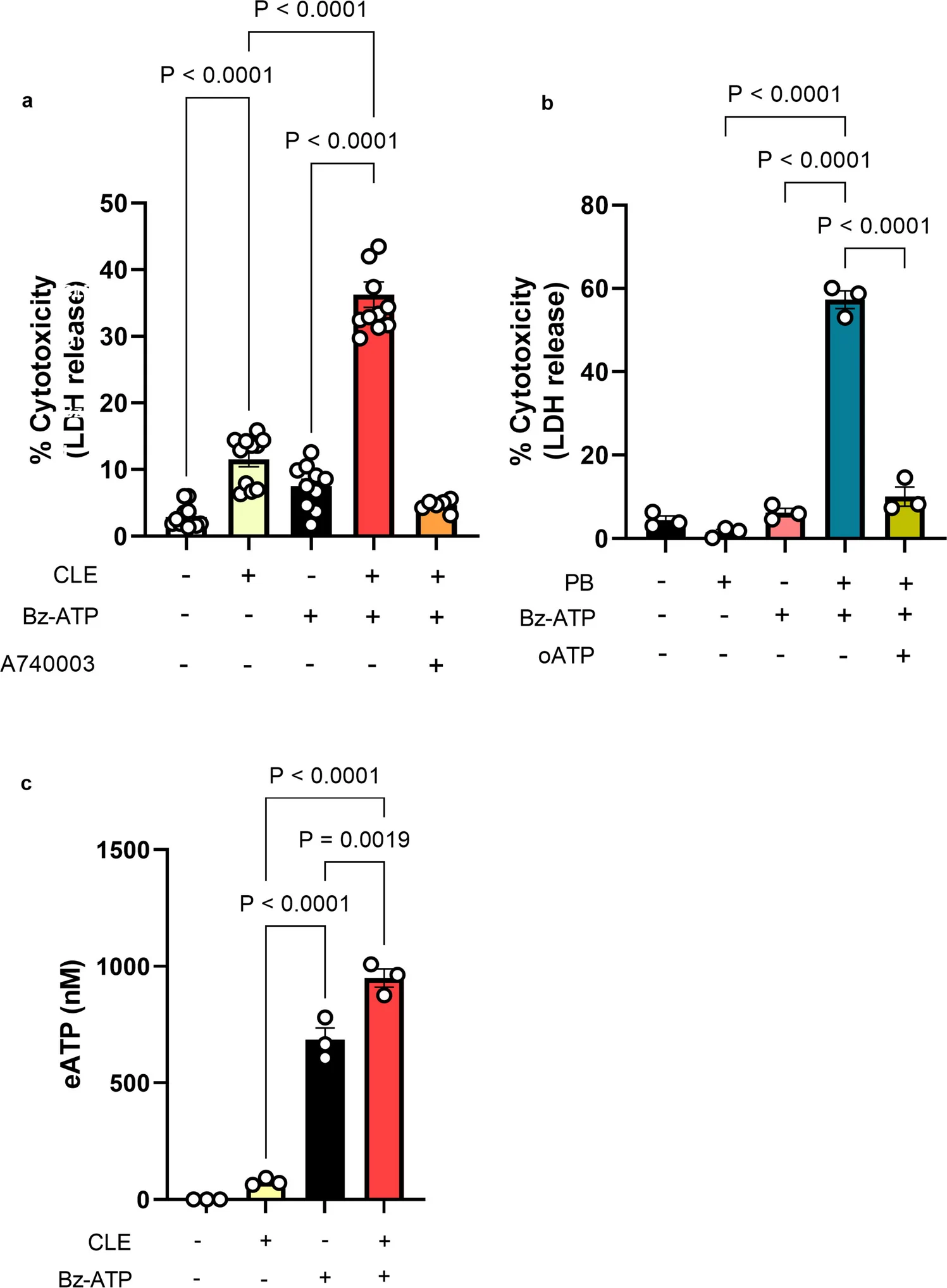
Positive allosteric modulators (PAMs) increase P2X7-dependent cytotoxicity and eATP release in murine tumor cells. **a**, **b** B16F10 WT cells (2.0 × 10^4^ cells/well) were seeded into 24-well plates. After 24 h, cells were treated overnight with Bz-ATP (300 µM), CLE (25 µM), or PB (10 µg/mL), in the presence or absence of the P2X7 antagonists A740003 (10 µM) or oATP (300 µM) as shown in the figure. Supernatants were collected for LDH quantification (see “Materials and methods”). Maximum LDH release was determined lysing control untreated cells (positive control) whereas culture medium served as negative control. Cytotoxicity (%) = (sample test−negative control)/(positive control−negative control) × 100. **c** B16F10 WT cells (8 × 10^3^ cells) were maintained in complete RPMI-1640 medium and treated for 30 min with CLE (25 µM) and/or Bz-ATP (300 µM). Levels of eATP in supernatants were measured by adding 100 µL of Luciferase/Luciferin (see “ Materials and methods”). CPS (counts per second) were converted into ATP concentration (nM) using an ATP standard curve. Data represent the mean ± SEM of at least three independent experiments performed in triplicate. Statistical analysis was performed using one-way ANOVA followed by Tukey’s multiple comparisons test. Results were considered statistically significant when *p* < 0.05

Treatment with both CLE and Bz-ATP resulted in significantly higher release of intracellular LDH compared to either stimulus alone, and P2X7 inhibition by A74003 treatment abolished this effect (Fig. 5a). Similar results were observed when an alternative P2X7 PAM, PB (10 µg/mL), and the P2X7 antagonist oATP (300 µM) were used (Fig. 5b). Consistently, B16F10 WT cells treated for 30 min with CLE and Bz-ATP released significantly higher amounts of eATP compared to separate treatments (Fig. 5c). Altogether these results demonstrate that CLE-induced mitochondrial damage, cell necrosis, and eATP release are strictly P2X7-dependent.

### Macrophages are sensitized to release IL-1β via P2X7 positive allosterism

Macrophages are essential components of the innate immune responses, playing key roles in antigen presentation, cytokine release, and other fundamental processes in inflammatory cell recruitment. Extensive literature indicates that P2X7 is required for maturation and release, via inflammasome-dependent pathway, of IL-1β and IL-18, cytokines critical for the process of pyroptosis [39, 40].

Peritoneal macrophages from wild-type (WT) or P2X7 knockout (P2X7 KO) C57Bl/6 mice were primed with LPS (1 µg/mL) for 2 h and subsequently treated with CLE (20 μM) and/or ATP (500 μM) for 30 min. In these experiments, the CLE concentration was selected based on a cytotoxicity assay at various dosages (Supplementary Figure 2 b–f), while the subthreshold ATP concentration was chosen because, on its own, it was insufficient to trigger marked activation of the NLRP3 inflammasome and the consequent release of IL-1β.

**Fig. 6:**
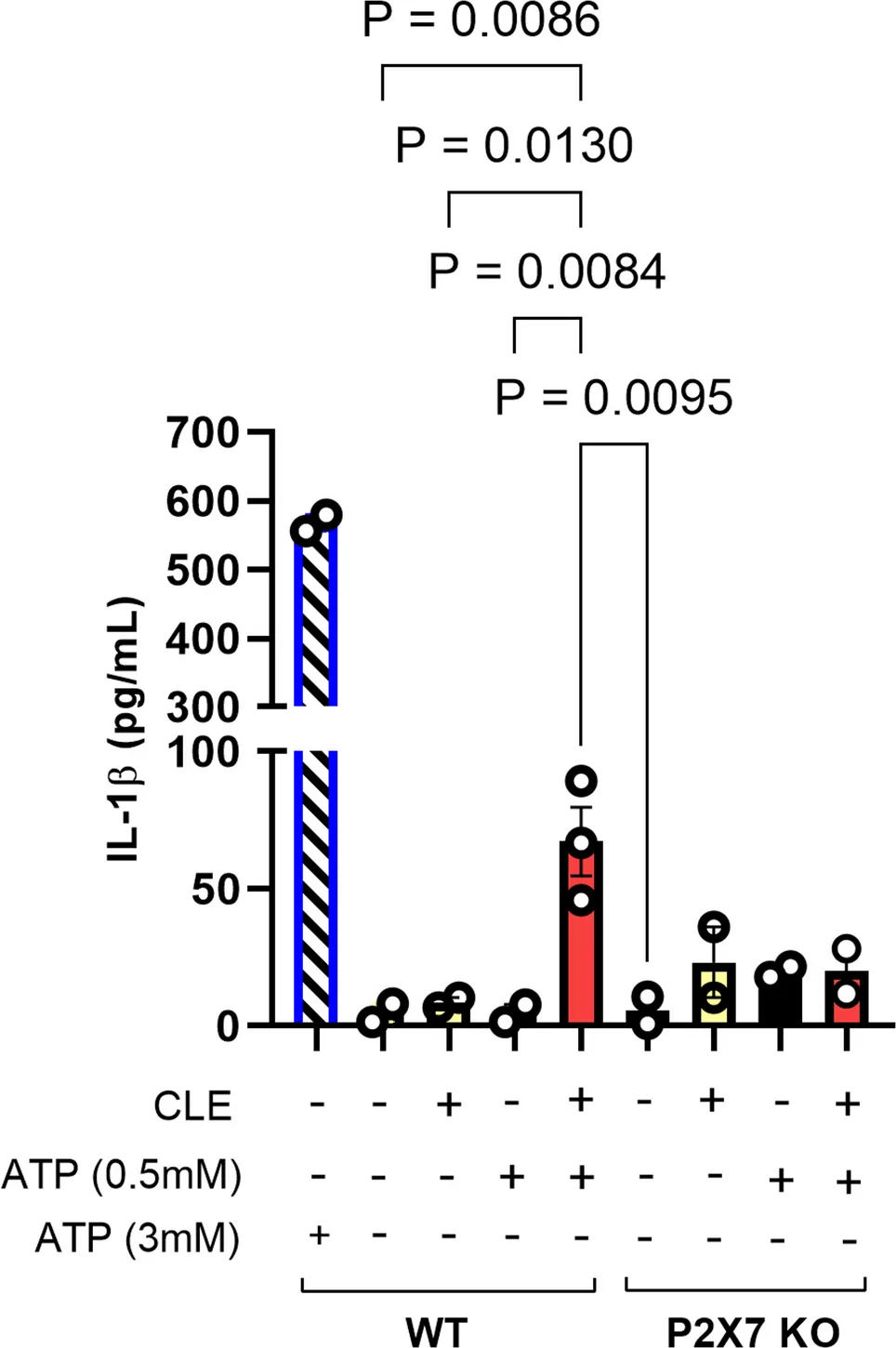
CLE increases P2X7-dependent macrophage activity. Peritoneal macrophages from WT and P2X7 KO C57Bl/6 mice were seeded at the density of 3.0 × 10^5^ cells into 24-well plates and cultured in complete DMEM. After 1 h, the cultures were gently washed twice with PBS to remove nonadherent cells. Adherent macrophages were primed for 2 h with LPS (1 µg/mL) and stimulated for 30 min with CLE (20 µM) and/or ATP (500 µM). Stimulation with ATP 3 mM alone was used to obtain the maximal IL-1β release. Supernatants were collected and, after centrifugation, IL-1β levels were measured by ELISA (see “ Materials and methods”). Data represent the mean ± SEM from three independent experiments. Statistical analysis was performed using one-way ANOVA followed by Tukey’s multiple comparisons test. Results were deemed statistically significant when *p* < 0.05

As shown in Fig. 6, macrophages from WT C57Bl/6 mice treated with both CLE (20 µM) and ATP (500 µM) released significantly higher amounts of IL-1β compared to macrophages treated with ATP alone. Stimulation with a maximal ATP concentration (3 mM) was used as a positive control to elicit maximal IL-1β release from these cells. Additionally, macrophages derived from P2X7 KO C57Bl/6 mice released comparable amounts of IL-1β regardless of ATP stimulation demostrating that CLE effectively enhanced IL-1β secretion through P2X7-dependent mechanisms. By acting as a PAM that enhances P2X7 receptor sensitivity, CLE effectively transforms an otherwise ineffective (subthreshold) dose of ATP into a potent activation signal, resulting in abundant IL-1β secretion.

## Discussion

The present study identifies clemastine fumarate (CLE) as an effective positive allosteric modulator (PAM) of the P2X7 receptor in both tumor and immune cells and provides evidence that pharmacological potentiation of P2X7 enhances membrane permeabilization, chemotherapeutic uptake, eATP release, mitochondrial alterations, and inflammasome-dependent cytokine release. These results further support the concept that controlled amplification of P2X7 signaling could be exploited therapeutically, turning a receptor frequently associated with tumor progression into a cytotoxic vulnerability for neoplasms overexpressing P2X7 [14]. P2X7 is unique among P2X receptors because of its dual gating properties. While short-term stimulation induces cation-selective channel opening, sustained or intensified activation promotes the formation of a large, nonselective macropore permeable to molecules up to ~ 900 Da [9].

In the present study, CLE significantly potentiated Bz-ATP-induced Ca^2^⁺ influx and ethidium bromide uptake in P2X7-expressing B16F10 mouse melanoma and HEK293-hP2X7 human cells, effects that were abolished by receptor antagonism or genetic deletion. These results demonstrate that CLE enhances both the ion channel and macropore functions of P2X7 in a strictly receptor-dependent manner, consistent with an allosteric mechanism that increases agonist efficacy and/or stabilizes the open conformation of the receptor. These data are consistent with previous studies demonstrating CLE-dependent P2X7 potentiation across different pathophysiological settings [29], including response to infections [41].

Of note, potentiation of macropore formation translated into increased intracellular accumulation of the anthracycline daunorubicin (DN). The ability of P2X7 to facilitate the uptake of otherwise poorly permeant chemotherapeutics has previously been described in hematologic malignancies and represents a distinctive pharmacological opportunity [35]. Our data extend this concept to melanoma cells and show that CLE markedly enhances DN nuclear accumulation in association with Bz-ATP. These findings indicate that P2X7 potentiation can functionally increase tumor cell permeabilization to cytotoxic agents. In the ATP-rich TME, where sustained eATP concentrations are frequently detected [4], pharmacological amplification of P2X7 activity may therefore lower the threshold for chemotherapeutic efficacy, potentially allowing dose reduction while preserving cytotoxicity. It remains to be tested whether CLE, together with chemotherapeutics, will have an effect on shifting human P2X7 isoform expression, as demonstrated in acute myeloid leukemia [35]. This will be of great interest, given the increasing awareness of the protumoral role of the human P2X7 isoform B in therapy resistance and cancer progression across different malignancies, including melanoma [35, 42–45].

Beyond drug uptake, CLE-mediated potentiation of P2X7 affected mitochondrial architecture and cell viability. Costimulation with CLE and Bz-ATP induced marked mitochondrial structural alterations typically associated with bioenergetic stress and execution of regulated cell death pathways [46]. P2X7-dependent mitochondrial remodeling has been linked to sustained Ca^2^⁺ influx, K⁺ efflux, and secondary metabolic perturbations [36, 37, 47, 48]. In our model, these changes were paralleled by increased LDH release, confirming the loss of plasma membrane integrity and cell death. The absence of these effects in P2X7-null cells or in the presence of selective antagonists underscores the receptor specificity of CLE action. The observation that CLE enhanced eATP release further suggests the existence of a feed-forward loop in which P2X7 activation promotes additional ATP efflux, thereby amplifying purinergic signaling within the TME [49]. Such amplification could reinforce local cytotoxic responses in tumor cells overexpressing P2X7, while simultaneously modulating immune cell function. In line with this, current evidence suggested that P2X7 stimulation remodeling the inflammatory landscape results in the activation of proinflammatory pathways [50]. Consequently, CLE emerges as a promising tool for reshaping immune responses in these settings.

In macrophages, CLE potentiated ATP-induced IL-1β secretion in a P2X7-dependent manner, shifting a subthreshold-to-threshold ATP concentration. Given that K⁺ efflux downstream of P2X7 is a canonical trigger for NLRP3 assembly, CLE-mediated positive allosterism likely lowers the activation threshold required for inflammasome engagement. Although we did not directly assess gasdermin D cleavage, increased IL-1β release together with enhanced membrane permeabilization is consistent with augmented pyroptotic signaling [51]. In addition, it was recently found that CLE with ATP causes pyroptosis of pluripotent stem cell–derived human oligodendrocytes and that an antagonist of P2X7, which is strongly expressed on oligodendrocytes and myeloid cells, blocked these CLE toxic effects [52].

In the context of cancer, induction of pyroptosis may reshape the immune landscape by releasing proinflammatory cytokines and DAMPs, and CLE-driven P2X7 potentiation may coordinate immunogenic inflammatory signaling with tumor cell permeabilization [53, 54].

These findings contribute to the ongoing reappraisal of P2X7 as a therapeutic target in oncology [3, 18]. While chronic, low-level P2X7 activation can sustain tumor growth and metabolic fitness, robust or pharmacologically amplified activation shifts the balance toward cytotoxicity. Positive allosteric modulation represents a strategy to exploit the high P2X7 expression characteristic of many tumors without indiscriminately activating the receptor in normal tissues. CLE, an FDA-approved antihistamine drug with well-characterized pharmacokinetics, offers a compelling candidate for drug repurposing. Its established clinical safety profile may facilitate translational development, particularly in combination regimens with chemo- or immune-therapies [30].

Some limitations warrant consideration. The extent to which CLE enhances P2X7 activation under usual eATP concentrations within the TME remains to be fully defined. Furthermore, given that IL-1β and IL-18 are known to share upstream activation mechanisms involving NLRP3 and caspase-1, it would be interesting in a future study to evaluate whether CLE treatment also enhances IL-18 release. This would further reinforce the importance of CLE in antitumor immunity. Although our data strongly support inflammasome potentiation, further studies assessing caspase-1 activation and gasdermin-D cleavage will be necessary to formally establish pyroptosis as the principal mechanism of CLE-enhanced cytotoxicity [51, 55]. However, our data showing increased CLE-dependent eATP release point toward this direction [56]. Finally, the balance between tumor cell death and the potential exacerbation of protumorigenic inflammation requires careful evaluation across different tumor contexts [45, 57]. Currently, we do not have direct structural evidence resolving the exact binding site of CLE to mP2X7. Nevertheless, results of patch-clump experiments from Norenberg et al. [29] indicate that CLE modulates hP2X7 activity by binding to the extracellular side of the transmembrane channel. Based on these results, our hypothesis of a direct allosteric interaction is supported by the rapid kinetic onset of the PAM effect on mP2X7, similar to what was observed for the hP2X7 receptor. Future structural investigations may resolve this question.

**Fig. 7:**
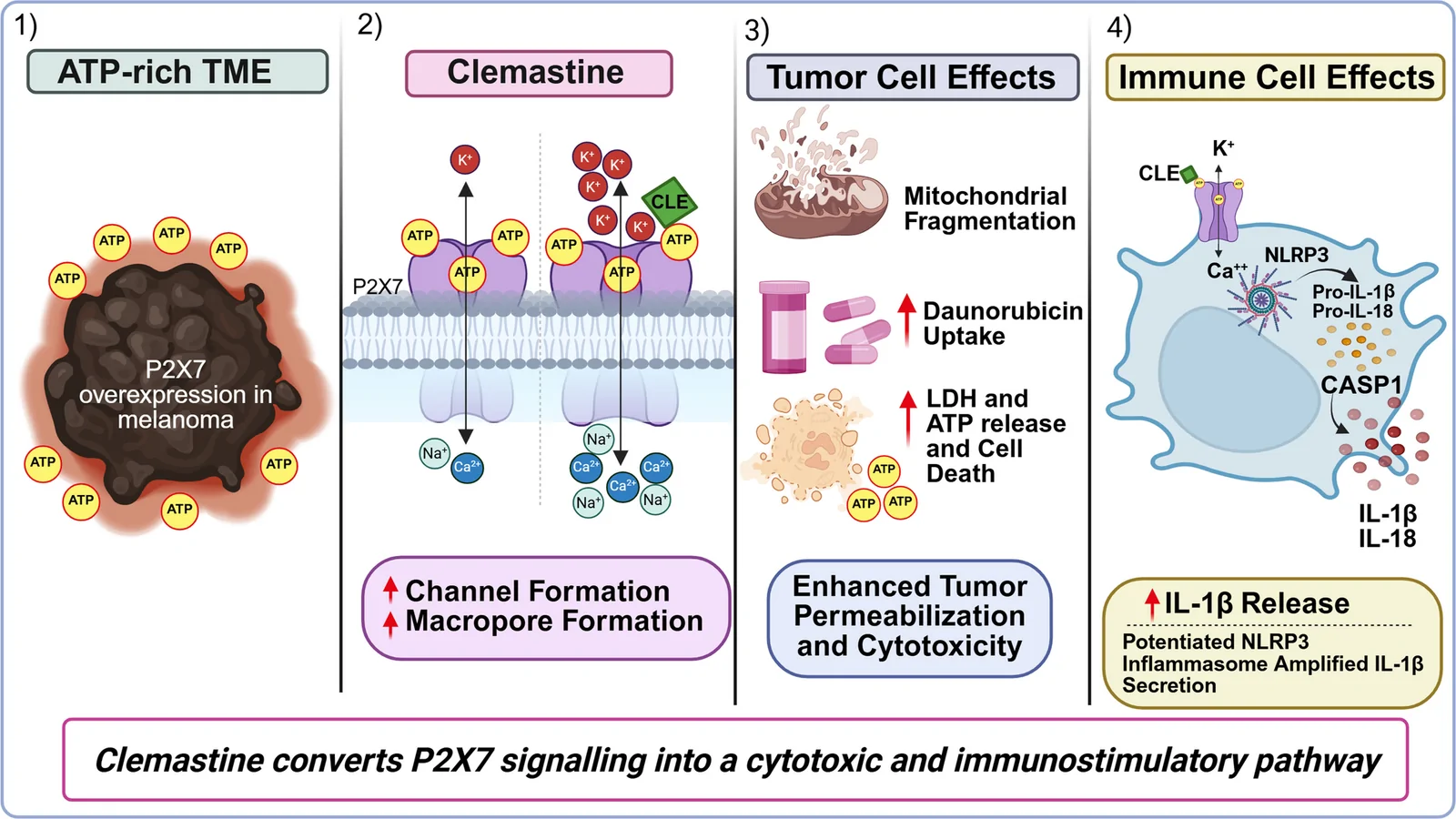
Graphical abstract. The tumor microenvironment (TME) is an ATP-rich niche where eATP at elevated concentrations acts as a critical signaling molecule at the P2X7 receptor that is overexpressed in melanoma cells. CLE acts as a positive P2X7 receptor modulator in B16F10 WT cells, where it significantly enhances P2X7 function, facilitating increased calcium influx and macropore formation (**2**), as well as mitochondrial structural alterations which collectively result in heightened DN uptake and cytotoxicity (**3**). Furthermore, in LPS-primed macrophages, the synergistic activity of CLE and ATP triggers the NLRP3 inflammasome pathway leading to significantly enhanced IL-1β secretion (**4**). Together, these results highlight the potential of CLE as a promising candidate for drug repurposing in the field of cancer immunotherapy

In conclusion, our results demonstrate that CLE acts as a potent PAM of P2X7, enhancing ion channel activity, macropore formation, chemotherapeutic uptake, mitochondrial disruption, and inflammasome-dependent cytokine release (Fig. 7). By promoting membrane permeabilization and cell death in P2X7-expressing tumors, CLE may convert purinergic signaling from a tumor-supportive pathway into a therapeutic liability. These findings provide a strong rationale for further exploration of P2X7 potentiation strategies as adjuvant approaches in cancer therapy**.**

## Supplementary information

Below is the link to the electronic supplementary material.

Fig. 8 Supplementary Figure 1 (S1). Evaluation by Western blot of P2X7 knockout by CRISPR/Cas9 in B16F10 cells. a,c Representative Western blot analyses of P2X7 protein expression in wild-type (WT) and CRISPR/Cas9-targeted knockout (P2X7^−/−^) B16F10 cells. Whole-cell lysates were immunoblotted using rabbit polyclonal anti-P2X7 receptor antibodies, directed against either the extracellular domain (Alomone Labs, cat no. APR-008), or the C-terminal domain (Sigma cat no. P8232) of the P2X7 receptor. Protein bands were visualized by an enhanced chemiluminescence (ECL) detection system and captured with a ChemiDoc™ Imaging System (Bio-Rad) or with a LI-COR blot scanner (Biosciences). Densitometry was performed using Image Lab Software (ChemiDoc system) or Image J (Li-COR system). The P2X7 receptor was identified as a characteristic band at approximately 68–75 kDa, representing the mature protein, while β-actin was used as the internal loading control. b,d Densitometric evaluations of the P2X7/β-actin bands from separate western blots. Comparisons were performed using the Student’s *t*-test. Results were considered statistically significant when *p* < 0.05. (PNG 285 KB)

ESM 1 High Resolution Image (TIF 697 KB)

Fig. 9 Supplementary Figure 2 (S2). Evaluation of cytotoxicity at different CLE concentrations. a B16F10 WT cells or b-f peritoneal macrophages from C57Bl/6 mice were plated as described in methods and treated overnight with different CLE concentrations (10–80 µM). LDH content was measured in supernatants harvested after plate centrifugation at 1100 rpm for 5 min. Maximum LDH release was determined lysing control untreated cells (positive control) whereas culture medium served as negative control. Cytotoxicity (%) = (sample test-negative control)/(positive control-negative control) × 100. Data, shown as means from three independent triplicates, were analyzed by one-way ANOVA and Tukey’s test. Results were considered statistically significant when *p* < 0.05. Photographs of cultured murine macrophages treated with different CLE concentrations (10, 25 and 50 µM) were acquired using a Leica inverted microscope at 40 × magnification. Scale bars are displayed in all photographs. (PNG 1.42 MB)

ESM 2 High Resolution Image (TIF 2.28 MB)

## Data Availability

No datasets were generated or analysed during the current study.
